# A Systematic Review of the Effectiveness of Virtual Reality-Based Interventions on Pain and Range of Joint Movement Associated with Burn Injuries

**DOI:** 10.3390/jpm12081269

**Published:** 2022-07-31

**Authors:** Elisa María Garrido-Ardila, María Santos-Domínguez, Juan Rodríguez-Mansilla, Silvia Teresa Torres-Piles, María Trinidad Rodríguez-Domínguez, Blanca González-Sánchez, María Jiménez-Palomares

**Affiliations:** 1 ADOLOR Research Group, Department of Medical-Surgical Therapy, Medicine Faculty and Health Sciences, Extremadura University, 06006 Badajoz, Spain; egarridoa@unex.es (E.M.G.-A.); blgonzalezs@unex.es (B.G.-S.); mariajp@unex.es (M.J.-P.); 2 Alfa Córdoba Center, 27, Teruel Street, 14011 Córdoba, Spain; mariasantosdominguez.to@gmail.com; 3 Research Group in Immunophysiology, Department of Medical-Surgical Therapy, Faculty of Medicine and Health Sciences, University of Extremadura, 06006 Badajoz, Spain; storres@unex.es; 4 ROBOLAB Research Group, Medical-Surgical Therapy Department, Nursing and Occupational Therapy Faculty, Extremadura University, 10003 Cáceres, Spain; trdomin@unex.es

**Keywords:** virtual reality, therapy, burn, pain, range of motion

## Abstract

Background: Burns are mild or severe lesions produced in living tissue, due to the action of different agents. This pathology is considered the third cause of accidental death in the world by the World Health Organization. Among the most disabling sequelae in these patients, pain and range of motion have the greatest impact. A recommended tool to complement the treatment or management of the symptoms associated with burns is virtual reality. Objective: The objective of this study was to analyse the effectiveness of virtual-reality therapy for pain relief and the improvement of the range of joint movement in patients who have suffered burns. Methodology: This study is a systematic review conducted following the PRISMA statements. An electronic literature search was performed in the following databases: PubMed, Cochrane, Dialnet, Scopus and Science Direct. The inclusion criteria were: participants with burns in any part of the body, interventions with virtual reality with or without complementary treatment, studies in both Spanish and English, and outcome measures of pain and range of motion. Results: Finally, 10 studies were included in the review. The sample consisted of one pilot study, three randomized controlled clinical trials, one prospective randomized controlled clinical trial, one control group and treatment group trial, one interventional clinical trial and three comparative studies. The most commonly used assessment tools for pain were the graphic rating scale (GRS) and for range of motion the goniometer. The use of virtual-reality games significantly reduced pain scores during physiotherapy and occupational therapy treatments as well as in nursing care. The range of motion improved significantly during virtual-reality exercises performed during a physiotherapy treatment in 33% of studies included in this review. Conclusion: The results of the studies analysed in this systematic review suggest that the use of virtual reality for the management of pain and range of movement limitations associated with burn injuries could control these symptoms and decrease their negative consequences on the person.

## 1. Introduction

Burns are lesions produced in living tissues mainly affecting the largest organ of the human body, the skin. They are due to the action of different agents and can range from mild to severe lesions [1]. This pathology is considered by the World Health Organization (WHO, Geneva, Switzerland) as the third cause of accidental death in the world [1,2]. In Spain, approximately 1000 patients are admitted to the major burn units of referral hospitals each year [3]. Burn injuries account for 6–10% of the emergency department consultations [1,2].

The most common complications include pulmonary failure, acute renal failure, infection of the affected parts, sepsis or multiorgan failure [1]. If left untreated, all of these complications can lead to death. Those who survive usually have physical, functional, esthetical and psychological sequelae that interfere with the patient’s life [1]. One of the most disabling sequelae in these patients is pain, which likewise makes them adopt bad posture and leads to a decreased range of joint movement [4].

To avoid all these complications, a series of preventive measures based on intensive care must be implemented (mobilization and postural control) [4]. Medical treatment is usually aimed at preventing infection, promoting healing and avoiding retractions and sequelae. All medical treatments are effective for the survival of the person, but do not achieve a full and comprehensive recovery [3,5].

At present, burns have become a problem that affects a significant part of the world’s population, causing a multitude of changes, both physical and psychological, in the people who suffer from them, as well as altering their routines and the daily rhythm of their lives [3,6,7]. The complexity of these patients’ conditions implies that being treated by a single specialist will not be sufficient. Therefore, a multidisciplinary approach that allows the integration of the knowledge and experience of multiple experts in the field is recommended to provide adequate care. This approach would include surgeons, internist physicians, anesthesiologists, nurses, physiotherapists, occupational therapists, speech therapists, mental health and social workers, with the team leader being a surgeon expert in the area and with management skills, if possible [3,6]. This multidisciplinary approach is essential because the complex treatment that people affected by burns requires should aim for an optimal recovery of their function which will allow them to participate in society, psychologically and physically [3,6].

According to the scientific evidence, technological advances have come to the aid of the rehabilitation treatment of patients who have suffered burns, reducing pain during mobilization and increasing their motivation and participation in the whole process. Therefore, virtual reality has been considered as a recommended tool for these patients [8].

Virtual reality (VR) has become popular in clinical research studies as an innovative form of distractor technique of computer technology based on the use of computers and other devices that recreates life-like settings in a digitalized world [9]. It provides an opportunity for people to actively interact with this new environment in order to produce an appearance of reality that allows the user to have the sensation of being present in it [10].

This technique has been used for the management of pain and distress in a wide variety of known painful medical procedures. In addition, the technique seems beneficial for a wide age range of paediatric patients and can be exceptionally well-adjusted for paediatric medicine, a difficult-to-manage population in clinical burn situations [11].

VR is therefore a technology with many interactive possibilities, especially in an immersive approach related to 3D images and sound which also offers the possibility of encompassing other human senses. In addition, perceptive VR can be immersive and non-immersive [9]. Virtual reality is a simulation technology that can be immersive, allowing the user to interact with a three-dimensional (3D) image generated by the computer. The scenes are primarily visual and manipulated through helmets, gloves or other devices that capture the rotation and position of different parts of the body. The interactivity of virtual reality is possible thanks to a tracking system that tracks the movements of the patients and allows the user to feel involved in the virtual environment, providing a sensation of being in an environment as if it were real [9]. Non-immersive methods normally are less interactive, so are often created by 2D interaction devices such as keyboards and mice without fully immersing in the environment [12]. They do not require the integration of new devices [9].

Virtual reality has three necessary elements involved in motor learning: repetition, sensory feedback, and subject motivation [13]. As plasticity is practice-dependent, repetition enhances the learning of motor and functional skills. Virtual environments are capable of providing massive and intensive sensorimotor stimulation and feedback, essential to induce brain reorganization. Patient motivation is achieved by focusing on the different activities that present the subject’s therapy in a pleasant and engaging way [14].

Likewise, VR has shown to be an effective technique for the management and control of pain caused by burns, by provoking beneficial effects related to the reduction of the sensory and emotional component involved or by improving the strength and range of joint movement [15,16,17]. Different studies [11,18,19] have shown its efficacy in the treatment of acute pain using a technique which consists of performing VR-based behavioural interventions to mitigate the pain experienced by patients undergoing painful medical procedures, such as burn injuries. VR aims to distract the patient by diverting his or her attention away from pain signals [20]. Similar studies have demonstrated its efficacy in reducing pain and anxiety in paediatric patients when used as a distraction before a medical procedure [21]. These results coincide with those presented by Scapin et al. in 2018 [8], who added to the benefits of VR use in burn patients the improvement of the wound epithelialization and reduction of the hospitalization time. However, neither of these studies showed results regarding the range of movement.

The main objective of this systematic review was to analyse the effectiveness of virtual-reality therapy for pain relief and the improvement of the range of joint movement in burn patients.

## 2. Materials and Methods

### 2.1. Study Design

This systematic review was carried out following the PRISMA statement [19], and was registered in PROSPERO with the registration number CRD42021250965.

### 2.2. Search Strategy

To identify relevant studies, the search was performed in the following databases and search engines: PubMed, Cochrane, Dialnet, Scopus and ScienceDirect. The keywords used were virtual reality, therapy, burn patients, pain and range of motion. These keywords were entered in Spanish when required by the database. The keywords were combined with the Boolean AND operators. Thus the search equation used was: “Virtual Reality AND therapy AND pain AND burn patients AND range of motion”. translated into Spanish in those databases that required it. The syntax of combined descriptors in the scientific database search can be found in Table 1.

### 2.3. Eligibility Criteria

The selection criteria were established following the PICO model (population, intervention, control, and comparison and outcomes). The inclusion criteria were:

- Type of participants: subjects with burns in any part of their body showing decreased range of joint movement and pain on rehabilitation. No age range or gender was limited.
- Type of intervention: virtual-reality interventions with or without additional or complementary treatment.
- Study type: randomized controlled trials, quasi-experimental studies, studies with experimental and control groups and clinical studies. The language of the studies was established as English or Spanish. Due to the specificity of the topic and the lack of related scientific production, the date of publication was not limited in the searches.
- Outcome measures: pain and range of joint movement.

The exclusion criteria were:

- Meta-analyses, studies with less than two treatment sessions, study protocols, and qualitative descriptions.
- Studies with only one participant.

### 2.4. Selection of Studies

The pre-selection of the studies was made taking into account that they were within the proposed object of study. This selection was carried out by reading the summary of the studies and excluding those that did not meet the established criteria. The full texts of the studies that did meet the inclusion criteria were reviewed, analysed and included in the systematic review.

The following data were obtained from the studies included in the review: characteristics of the sample, study design, description of the intervention and of the control and experimental groups, outcome measures and study results. These data were compiled in a standard table. We also collected the data and assessed the methodological quality of the studies.

All potential full-text articles were independently retrieved and evaluated by two reviewers. The level of agreement between the two reviewers was not specifically calculated. However, any disagreement on inclusion or exclusion of the full-text articles was discussed and resolved.

### 2.5. Methodological Quality Analysis

The methodological quality of the studies was assessed using the PEDro (Physiotherapy Evidence Database) scale. This scale has 11 items that can be answered with “yes” (Y) or “no” (N). The total range of scores is from 0 to 10 according to a methodological quality from low to excellent. The results obtained on the scale are considered to be of high quality if the score is above 5 (6–8: good, 9–10 excellent); of moderate quality if the score is between 4 and 5 (regular quality study); of low quality if the score is below 4 (poor quality study).

As the first item is related to the external validity and is not used to calculate the score obtained, the maximum score is 10. Items 2 to 9 aim to assess if the study has sufficient internal validity and items 10 and 11 analyse whether the statistical information is adequate to understand the results [22].

### 2.6. Risk of Bias Analysis

The risk of bias [23] was calculated for each included study, referring to the following type of bias: selection bias, performance bias, detection bias, attrition bias, information bias, and other biases. In this assessment, 7 criteria were assessed: 1 = random sequence generation (selection bias). 2 = allocation concealment (selection bias). 3 = blinding of participants and personnel (performance bias). 4 = blinding of outcome assessment (detection bias). 5 = incomplete outcome data (attrition bias). 6 = selective reporting (reporting bias). 7 = other bias. The risk of bias and the study quality were calculated by a single reviewer.

## 3. Results

**Figure 1:**
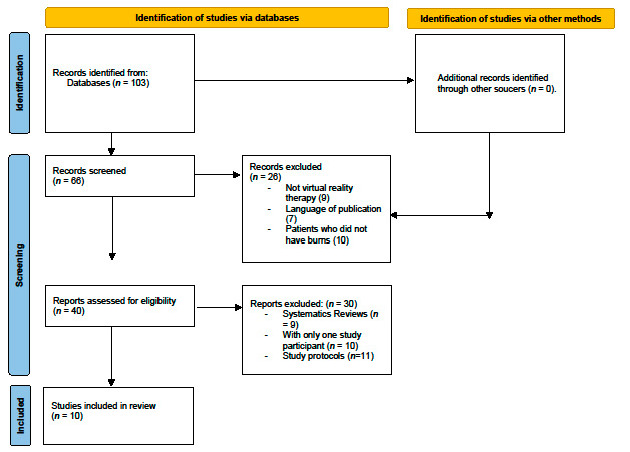
PRISMA flowchart.

A total of 103 studies were obtained from the search of all the databases; duplicate records were excluded and 65 studies were selected. The final sample consisted of 10 studies in the end. The study selection process is shown in the PRISMA flow chart (Figure 1).

### 3.1. Sociodemographic Characteristics

Regarding the sociodemographic characteristics of the participants of the studies, the ages ranged from children of 5 years to adults of 78, with the mean age of all studies [17,21,24,26,27,29,30,31,32,33] being 23.44. In terms of sample size, the number of participants ranged from 23 [20] to 66 [24]. Considering all the samples, the studies had a total number of participants of 459.

### 3.2. Methodology of the Studies

Regarding the methodology of the studies analysed, the sample consisted of a pilot study, three randomized controlled clinical trials, a prospective randomized controlled clinical trial, a control group and treatment group trial, three comparative studies and one interventional clinical trial. All papers were published between 2009 and 2022. Table 2 shows the main characteristics of the studies included in this systematic review.

#### 3.2.1. Interventions

All of the reviewed studies [17,20,23,24,25,26,27,28,29,31] applied the same type of intervention: immersive virtual reality. Five of the studies combined VR with pharmacological therapy [25,26,27,28,29] by taking analgesics before and during treatment. Other authors combined VR sessions with physical range-of-motion therapies of the affected joints [17,20,23,24,25,26,29,31].

Regarding the duration of the virtual reality-based interventions, it varied among the studies. Radwan et al. [31] conducted an intervention that lasted 60 min, 30 min of traditional therapy followed by Wii therapy for 30 min. In the study by Kamel et al. [17] the treatment lasted 50 min, 3 days a week for 8 weeks with motion detection games and interactive video games with the Xbox. In the research of Soltani et al. [25], participants performed range-of-motion exercises while watching with virtual-reality goggles and moving the mouse to play a game of “snowballs” with the hand that was not doing exercises. The average treatment duration was approximately 3 min. Carrougher et al. [26] carried out a treatment programme of 10-minute sessions of exercises with and without virtual reality. As for Faber et al. [27], the intervention consisted of up to seven virtual-reality sessions performed during the wound care of the participants but the duration was not specified. Hoffman et al. [28] alternated 5 min with virtual reality and another 5 min of treatment without virtual reality while the staff cared for the wounds. Schmitt et al. [29] conducted a study in which the sessions lasted between 6 and 20 min and were divided into two consecutive parts of identical duration of 3–10 min each, using pharmacological therapy, virtual reality and physiotherapy. In the study by Yohannan et al. [20], the treatment consisted of three consecutive sessions of standardized therapy consisting of a passive range of motion exercises and a combination of predetermined and joint-specific exercises for 15 min. This was followed by an additional 15 min of Wii play. Finally, Lozano et al. [24] participants received standardized physiotherapy treatment and Xbox Kinect sessions. Treatment sessions lasted between 30 and 45 min and were performed at least once or twice a day. This same study conducted a minimum of 2 weekly Xbox Kinect sessions of 15–30 min each. Finally, in the study conducted by Lee et al. [30], 30 min robot-assisted gait-training (RAGT) sessions were performed 10 times for 2 weeks. Robotic training alone, without VR, was performed for 15 min, and VR and RAGT were performed simultaneously for 15 min each.

#### 3.2.2. Professionals

When analysing the use of virtual reality as an intervention tool, we can observe that none of the studies specified whether the professionals that guided the treatment had been trained to be able to administer virtual-reality therapy. As for the professionals who performed the therapy, in six of the studies [17,24,25,26,29,31] a physiotherapist conducted both the virtual-reality therapy and the complementary therapy. In two of the trials [16,29], an occupational therapist also carried out both virtual-reality therapy and the others. Yohannan et al. [20] and Soltani et al. and Lee et al. [25,30] did not specify the professionals involved in the intervention as they only addressed them as “therapists” without going into further detail. In the study by Hoffman et al. [28], it was the nursing staff who carried out the treatment, while Kamel et al. [17] did not specify.

#### 3.2.3. Outcome Measures and Assessment Tools

The most important outcome measures analysed by the studies were the range of motion and pain, since both were assessed in four studies included in this review [20,25,26,29]. Pain was analysed as a single outcome measure in the studies by Kamel et al. [27], Hoffman et al. [28] and Lee et al. [30], while the range of joint movement was measured as a single measure in three of the studies [17,24,31].

The most commonly used assessment tools for the evaluation of pain and range of joint motion were the graphic rating scale (GRS) of pain and the goniometer, appearing in seven of the studies [17,20,24,25,26,28,29,31]. Both the GRD and the goniometer were used in the trials by Carrougher et al. [26] and Soltani et al. [25], while the goniometer was used in five of the studies [17,20,24,25,26] and the pain GRS in four [25,26,28,29]. For the assessment of pain, the visual analogue thermometer (VAT) or the visual analogue scale (VAS) was also used in two studies [20,27] and in the study by Hoffman et al. [28] the Pain Catastrophizing Scale for Children (PSC-C) was used. In the Lee et al. study [30], the portable functional near-infrared spectroscopy (fNIRS) measurement system, which was attached to the head with elastic straps inside a plastic cap, was used. This system uses 24 laser sources to measure cerebral blood flow (CBF) analysis in the prefrontal cortex throughout the intervention.

### 3.3. Results of the Studies

In the following subsection, the results obtained in the studies in relation to the variables of interest, range of joint movement and pain, are described. The study by Yohannan et al. [20] showed that the Wii group experienced less pain over time than the control group while the active range of motion did not improve significantly in the Wii group. Carrougher et al. [26] had evidence that virtual reality reduced all pain scores. In contrast, the range of motion was slightly greater in the VR group, but it did not reach statistical significance. In the study conducted by Schmitt et al. [29], the subjects reported a significant decrease in pain scores during VR on the first day of the study. The improvements in analgesia and affective pain were maintained with the repeated use of virtual reality in multiple therapy sessions. The peak range of motion was not different between treatments, but it was significantly greater after the second treatment session. Based on the results of the trial by Soltani et al. [25], participants showed lower mean pain scores during VR than without VR, in terms of worst pain, pain discomfort and time spent thinking about the pain. However, the patients did not show a greater range of motion during VR.

In the study by Hoffman et al. [28], VR significantly reduced “worst pain” scores during wound care processes in intensive care units. Faber et al. [27], found statistically lower pain rates when patients were in VR in the first few days. In addition, from the fourth to the sixth day, they were aware that VR continued to reduce pain when used repeatedly.

The use of the Xbox Kinect [24] was effective in improving the active range of movement between discharge and follow-up. In addition, Radwan et al. [31] concluded, based on their results, that the participants in the study group significantly improved their hand-to-head and hand-to-mouth function. In the hand-to-shoulder task, the range of movement improved significantly in both the study and control groups. However, the control group revealed a significant improvement only in the contralateral hand-to-shoulder task, while the hand-to-head and hand-to-mouth tasks did not change significantly. Lastly, in the study by Kamel et al. [17] there were no significant changes in the range of motion, grip strength and pinch strength between the Xbox group and the task-oriented training group. Conversely, there was a significant increase in palm pinch strength in the motion sensing, hands-free and gaming-device groups compared to the task-oriented training group after the intervention.

Regarding analgesic intake, we must highlight that in five of the studies [25,26,27,28,29] they were administered before VR treatment and before the intervention without VR. All studies used oral opioid analgesics and in only one trial [29], they were combined with oral benzodiazepine. Carrougher et al. [26], was the only study to have opioid equivalence as a measure, thus representing the amount of opioids received. The equivalence was slightly different, as the measure of opioid equivalence administered on the VR day was 0.87, whereas on the non-VR day, the measure was 1.04. Even if this measure was higher without VR, the difference was not statistically significant.

Finally, in the Lee et al. study [30], the mean VAS pain scores were significantly lower (*p* < 0.05) in the experimental group than in the control group. Moreover, oxyhaemoglobin in the prefrontal lobe significantly increased when robot-assisted gait training (RAGT) was performed with VR and the results of Hb02 analyses performed in the prefrontal cortex indicated a significant activation related to VR during RAGT, compared to the control group.

### 3.4. Methodological Quality of the Included Studies

Five of the studies [20,24,25,26,31] included in this review scored ≥6, indicating good methodological quality. Four studies [24,28,29,30] scored 5 and one study [27] scored ≤4, indicating medium quality. Randomization was performed in six of the studies [17,20,25,26,29,31] and concealed allocation was carried out in only one of the included studies [17]. In none of the trials were the subjects blinded while the assessors were blinded in only one study [17]. Table 3 shows the results of the methodological quality of the studies.

### 3.5. Risk of Bias

It should be noted that three of the selected studies [24,30,31] presented a low risk of selection bias since they were randomized, although only one of them [31] also presented allocation concealment. Regarding performance bias, almost all of them had a high risk, except for the study conducted by Lozano et al. [24], which had an unclear risk. Regarding detection bias, four of the articles included in this review [17,24,28,31] had an unclear risk with one of them [31] being a low risk as well. Regarding dissertation bias, all [17,20,23,24,25,26,27,28,29,31] were low risk. Table 4 shows the results on the risk of bias.

## 4. Discussion

This systematic review assessed the effectiveness of virtual-reality therapy in improving pain and range of joint movement in burn patients. The results suggest that the use of virtual reality in the treatment of burn patients is effective in decreasing pain and increasing range of motion during both wound-care procedures and physical rehabilitation.

No age limit was established in the eligibility criteria in order to have a greater variety of studies, taking into account that there are scarce studies on the subject. In our review, children and adolescents predominated in the studies analysed. This coincides with Tadín et al.[32] who showed that children and adolescents were the most affected by burns. In addition, in the systematic review by Castellanos et al. [33], 13 studies were selected and all the participants were children and adolescents with burns up to 18 years of age. This could be due to the fact that the younger people are familiar with and have knowledge of the technologies, and they are the ones who are unlikely to reject the treatment. On the other hand, it should be noted that older people may have the same feelings as adolescents as was shown in a virtual-reality programme [34] carried out for older people with an average age of 82 years. The authors saw that 100% of the participants liked the experience and said they enjoyed it. Of them, 95.8% would like to repeat the experience and 91.6% would like to do it frequently. Some 97.4% would like to try more VR experiences, especially to see familiar places they have not visited for a long time or places they have never had the opportunity to visit. Regarding the feelings and emotions they experienced, 93% felt a sense of reality during the immersion and 42.5% felt that the different scenarios evoked memories. Of them, 18.3% had feelings of familiarity and only 1.69% experienced some kind of emotion during the experience. This shows that VR can be just as effective and familiar for adults as it is for teenagers, and can be seen as an opportunity for them to revisit the past.

In terms of sample size, most of the analysed studies had a sample of more than 30 participants. This sample size can be considered adequate, but it would be desirable to have a larger number of samples in order to obtain more reliable or extrapolable to the population results. In contrast, a study conducted by Sharar et al. [35], which conducted a virtual-reality treatment, had a larger sample than the studies included in this review, using virtual reality for burn patients, with a total of 88 participants.

In relation to the burned area, no specific area or type or phase of burn was established due to the limited number of articles available and, therefore, the requirement of having burns in some part of the body was established as an inclusion criterion. Furthermore, no studies have been found in the literature with a specific type or phase of burns as inclusion criteria for the entire sample, which supports the criteria established in this review. We believe that there is a need for research studies that focus on a specific type and phase of burn, as not all burns will have the same pain and limitations in the range of motion and will depend on the affected parts of the body, type of burn and phases of healing.

The most commonly used assessment tool for pain was the graphic pain scale (GRS) which coincides with the study by Maani et al. [36] who used the scale to measure pain, including comments on the efficacy of the scale. The most commonly used tool for the range of joint movement evaluation was the goniometer. This was not the case with the study by Park et al. [37], who used the Fugl-Meyer upper extremity assessment (FMA-UE), a more standardized scale, although it has not been shown to have better reliability than the goniometer.

All interventions lasted between 60 min [25] and 5 min [28], which is consistent with the included studies discussed in the previous review [33], in which the interventions lasted between 10 and 55 min.

Regarding the side effects of virtual reality, none of the 10 studies included in this systematic review described any adverse or side effects of this therapy. This supports the use of virtual reality as the analysis of the interventions has mainly shown benefits.

The results obtained in the studies included in this systematic review have been analysed and are discussed by outcome measure (pain and range of movement) hereafter.

### 4.1. Virtual Reality and Pain

After reviewing the scientific literature, the evidence suggests that there is a relationship between virtual-reality therapy and pain reduction. VR helps to distract the person and situates him/her in a virtual world which decreases pain by distraction. Distraction has been used as a pain relief technique in different disciplines, such as nursing [38]. In this review, the most-used interventions for pain management were physiotherapy and occupational therapy, in addition to virtual reality. No other studies on burn patients were found in the literature to contrast this information. Two of the analysed studies [27,28] found significant improvements in pain during and after VR therapy. These results are consistent with those obtained in other research in patients undergoing surgical procedures [39]. It also coincides with a previous systematic review [40] that included 17 studies showing that the use of virtual reality or physiotherapy sessions in burn patients during the healing process could be an effective method of decreasing the intensity of pain and anxiety.

Furthermore, according to the study conducted by Carrougher et al. [26], virtual reality decreases medication intake which is closely related to pain levels. The study was based on virtual-reality therapy with the ‘SnowWorld’ game in addition to physiotherapy treatment and obtained significant results in pain relief. These results coincide with the study by Hoffman et al. [16], not included in this review due to eligibility criteria, who performed a virtual-reality intervention while performing physiotherapy exercises in burn patients. It also coincides with another study by Morris et al. [41] that carried out an intervention based on virtual-reality treatment, physiotherapy and pharmacological treatment with this type of pathology. Therefore, the three mentioned studies coincide in their results which indicate that VP improves pain.

Further research should be conducted to analyse whether virtual-reality therapy could be beneficial in decreasing analgesic intake and, therefore, could reduce medication side effects. In the study by Lee et al. [30], this therapy was used without the use of complementary analgesics and was the only pain relief treatment, which suggests that VR can be a solid nonpharmacological pain reduction technique for burn patients. These results are consistent with an intervention program developed for anxiety reduction in patients undergoing surgery [29], which showed that there was a significant decrease in medication intake for the experimental group with virtual-reality treatment.

It is important to highlight that the methodological quality of the studies included in this review that analysed the effect of VR in pain had a medium and good methodological quality. This supports the reliability of the studies’ results. Based on this and the results found, which relate virtual reality with distraction and pain reduction, virtual reality could be considered an effective therapeutic tool to be combined with other pain relief approaches and interventions.

### 4.2. Virtual Reality and Range of Movement

All seven studies that assessed range of movement [17,20,23,24,25,26,29,31] showed improvements of this variable. Furthermore, three of those studies [24,29,31] showed significant changes with virtual-reality treatment. These results coincide with those of Park et al. [37] who demonstrated significant results in a study related to virtual reality in stroke survivors, increasing the active range of motion in the treatment group.

Patients undergoing traditional physical therapy go through a series of sessions performing exercises to help improve the range of motion in the affected regions of the body due to illness or injury. However, patients find these tasks repetitive and boring and end up not completing the prescribed therapy program. In contrast, it has been shown that game-based therapy exercises have led to increased compliance rates [42,43]. Unlike traditional treatments, virtual reality allows an even higher degree of exposure to be achieved than would be possible in vivo, since the manipulation of a virtual environment makes it easier to emphasize those situations to be recreated; this is why the feeling of immersion is a key aspect [43]. Virtual reality-based physical therapy is one of the most innovative and promising recent developments in rehabilitation technology, but much research is still lacking. Although there is a wide range of virtual-reality therapies applied in various pathologies, few studies focus on burn patients. Virtual reality does not cure, but it is a good complement to other treatments for the optimal rehabilitation of the patient [43,44].

Regarding the methodological quality, one of the studies that assessed the range of movement was the one conducted by Kamel et al. [17], which obtained the highest score, translating into a good methodological quality. In addition, the study had a low risk of bias in almost all the criteria. This methodological quality supports the reliability of the results of the studies. Therefore, VR can be considered an effective method to increase the range of joint movement. It also allows a greater variety of therapeutic exercises, to which the aforementioned distraction factor is added. This could contribute to achieving greater joint ranges without the sensation of effort or the monotony of repetition.

### 4.3. Limitations of the Study

The fact that in the literature available there are very few experimental studies analysing the effectiveness of virtual reality in burn patients to address the measures of pain and range of motion may be considered a limitation of this review. The inclusion criteria established could have influenced the number of studies found and ultimately included in the review but the criteria were established to ensure maximum homogeneity of the studies and interventions. However, the results of the review showed that the studies were heterogeneous and treated different age groups. In addition, the studies conducted diverse interventions, assessed different outcome measures and used a variety of measurement tools, even though they met the inclusion criteria.

In terms of methodological quality, the studies included in this review scored between 4 and 8 on the PEDro scale. According to the PEDro interpretation guidelines, if studies scored at least 5 out of 10 they were considered to be of acceptable quality. Studies that scored around 4 did not include blinding of all patients, therapists and evaluators. Due to the nature of virtual-reality interventions, it is very difficult to have triple blinding, as a placebo cannot be used and the treatment provided is clear to the therapists. However, the evaluator could have been blinded in most studies.

### 4.4. Implications for Clinical Practice

We consider it important to highlight the implications for clinical practice of the results of our systematic review. Our results indicate that virtual reality-based interventions have many positive effects on burn patients, such as decreased pain related to the usual treatment [27]; increased range of movement [24]; increased fun and enjoyment in the sessions [25]; decreased feelings of vomiting and nausea [29]; and decreased medication intake [26]. This suggests that this therapeutic approach could be used as an adjunct to medical treatment. Therefore, we consider that the combination of VR with conventional therapies would be convenient for the rehabilitation of burn patients. We would also like to note that these results should be interpreted with caution, as the outcomes obtained in the studies analysed are very heterogeneous.

Regarding the professionals guiding the interventions, we observed that no study specified whether practitioners were trained in virtual reality or virtual-reality devices. The quality of the interventions carried out by virtual-reality professionals is probably based on the fact that they assess the specific needs of each patient and establish defined objectives and an individualized intervention plan. Moreover, as stated by Mantovani et al. [45], virtual-reality training can provide a rich, interactive and engaging educational context, thus supporting experiential learning.

We have also observed that VR interventions were carried out by occupational therapists, physiotherapists or nursing staff who do not specify whether they are specifically trained in this type of intervention. Virtual-reality therapy is increasingly being used by health professionals. Although some equipment has a high cost, they can usually be performed safely and effectively as an adjunct to the usual treatment. However, we consider that it would be more appropriate that the professionals in charge of the sessions were trained in virtual reality. This would ensure the correct development of the interventions, since they are the professionals who are specially, clinically and academically trained.

We suggest that future studies should have a larger sample size, homogeneous criteria such as burn type and stage, and adequate methodological quality. This would allow us to adequately analyse the effects of virtual-reality therapy, develop treatment protocols and extrapolate the results to other specific populations to improve the quality of life of patients.

## 5. Conclusions

The results of the studies analysed in this systematic review showed that virtual-reality therapy decreased pain in all the studies analysed and improved the range of joint movement in 33% of them. The studies showed that VR-based interventions have many positive effects on burn patients, including increased enjoyment of the sessions and a reduction in negative symptoms such as vomiting and nausea, as well as reduced medication intake.

These results suggest that the use of virtual reality for the management of pain and range of movement limitations associated with burn injuries could contribute to controlling these symptoms and decreasing their negative consequences on the person. However, due to the current scarcity of publications and the heterogeneity of the available studies, further research would be required.

## Figures and Tables

**Table 1:** Syntax of combined descriptors in the scientific database search.

Database	Syntax Adopted
PubMed	“Virtual Reality AND therapy AND pain AND burn patients AND range of motion”
Cochrane	“Virtual Reality AND therapy AND pain AND burn patients AND range of motion”
Dialnet	“Realidad Virtual AND terapia AND dolor AND quemados AND rango de movimiento”
Scopus	“Virtual Reality AND therapy AND pain AND burn patients AND range of motion”
Science Direct	“Virtual Reality AND therapy AND pain AND burn patients AND range of motion”

**Table 2:** Main characteristics of the studies.

Author	Country and Location	Age, Mean (SD), Median	Type of Study	Sample Size	Type of Intervention	Dose of Treatment	Outcome Measures/ Assessment Tools	Results
Yohannan et al. (2012) [20]	America Inc., Redmond, WA, USA	20–78 GE = mean 42.1 GC = mean 32.1	Comparative study	*n* = 28 (5 were eliminated) EG = 11 CG = 12	EG = active ROM therapy and Wii exercises. CG = therapy for active range of motion adapted by the therapist.	3 consecutive 15 min sessions of standardized therapy followed by an additional 15 min of Wii play.	Subjective assessment questionnaires developed. VAS goniometer.	The Wii group experienced less pain (× 0.97, *p* 0.07) than the control group (× 0.97, *p* 0.07) over time. Overall, trends with anxiety (× 0.1 l, *p* 0.77), AROM (× 0.55, *p* 0.81), function (× 0.38, *p* 0.43) and enjoyment (× 0.09, *p* 0.73) favoured the Wii group.
Lozano et al. (2018) [26]	Soweto (Gauteng) South Africa	5–9, mean 7	Control group and experimental group study	*n* = 66 CG = 35 XboxG = 31	XboxG = standard physiotherapy treatment and an Xbox Kinect. 1 or 2 times a day. CG = standard standard physiotherapy.	30 to 45 min treatment sessions performed at least 1 or 2 times a day.	Goniometer. activity scale for kids (ASK) Wong–Baker modified enjoyment rating scale (FACES)	The addition of Xbox Kinect in the treatment was effective in achieving higher AROM between discharge and follow-up (*p* < 0.01).
Radwan et al. (2020) [27]	Al-Kharj City, Riyadh, KSA. Saudi Arabia	7–12 GE = 9.52 ± 1.72 GC = 10.23 ± 1.75	Control group and experimental group study	*n* = 50 EG = 25 (3 excluded and 1 did not participate) EG = 21 CG = 25 (1 excluded and 1 did not attend) CG = 23	EG = 30 min of traditional physical therapy treatment followed by 30 min of Wii training. Approved games were bowling, baseball and tennis. CG = a conventional physical therapy program to maintain normal ROM.	30 min sessions of traditional therapy followed by 30 min of Wii therapy.	JTHFT	The study group significantly improved hand-to-head (*p* = 0.001, *p* < 0.001), hand-to-mouth (*p* = 0.001) and hand-to-shoulder (*p* < 0.001, *p* = 0.0018) movements compared to the control group.
Kamel et al. (2021) [17]	Cairo, Egypt	7–14, mean 10,70	RCT	*n* = 50 XboxG = 17 GTOT = 16 CG = 17	XboxG = conventional rehabilitation plus therapeutic games on Xbox. TOTG = conventional rehabilitation by increasing the time of the activity and modifying the time spent in the materials used. CG = conventional rehabilitation.	50 min sessions, 3 days a week for 8 weeks with motion detection games and interactive video games with the Xbox.	JTHFT DHI COPM Goniometer.	There were no significant changes in JTHFT performance and COMP performance, ROM, grip strength, and lateral and toe pinch strength between the Xbox group and the TOTG [*p* > 0.05].
Soltani et al. (2018) [25]	Seattle, WA, USA	15–66, mean 36	RCT	*n* = 39	Active ROM exercises while playing in virtual reality with the SnowWorld game.	Average treatment duration was approximately 3 min.	GRS Goniometer. Subjective evaluations.	No significant effect of VR on peak ROM was found when compared to order (No VR M = 59.0 ± 44.8 degrees; VR M ± 58.9 ± 43.6 degrees), t(37) *p* = 0.94 NS. Pain discomfort was also significantly lower during RV than during no RV (no RV M = 52.7 [SD = 28.8]; RV M = 29.3 [SD = 24.7], t (36) = 5.18, *p* < 0.001; SD = 27.44.
Carrougher et al. (2009) [26]	Seattle, WA, USA	21–57, mean 35	RCT and prospective study.	*n* = 41 (2 were withdrawn) *n* = 39	Virtual-reality therapy with the SnowWorld game plus physiotherapy and pharmacological therapy.	Ten-minute sessions of exercises with and without virtual reality. Total duration not specified.	GRS Goniometer.	VR reduced all GRS pain scores (worst pain, time spent thinking about pain, and pain discomfort) by 27, 37, and 37%. (worst pain, time spent thinking about pain, and pain discomfort by 27%, 37%, and 31%, respectively). The mean improvement in ROM was slightly slightly greater with the VR condition; however, this difference did not reach clinical or statistical significance (*p* = 0.243).
Faber et al. (2013) [27]	Netherlands.	8–57, mean 27.7.	Comparative study	*n* = 289 (253 excluded) *n* = 36	Virtual-reality therapy with the SnowWorld game.	Seven virtual-reality sessions performed during wound care of the participants.	VAT Subjective valuations.	VR reduced the amount of reported pain by more than one dressing change/wound debridement session per patient.
Hoffman et al. (2019) [28]	Galveston, TX, USA	6–17, mean 12.	Pilot study	*n* = 62 (14 excluded) *n* = 48	Virtual-reality therapy with SnowWorld game and nursing care.	Five min virtual-reality sessions alternated with another 5 min of treatment without virtual reality.	GRS Surveys to assess the user’s presence in the virtual world. PSC-C	VR significantly reduced the “worst pain” indices. during No VR = 8.52 (SD = 1.75) vs. during VR yes = 5.10 (SD = 3.27), t (47) = 7.11, *p* < 0.001
Schmitt et al. (2011) [29]	Seattle, WA, USA	6–19, mean 12.0 ± 3.9,	RCT	*n* = 54	Virtual-reality therapy with the SnowWorld game more physiotherapy to increase range of motion.	Sessions of 6 to 20 min divided into two consecutive parts of identical duration (3–10 min each) over 5 days	GRS Subjective assessments. Goniometer.	The GRS assessments of cognitive pain (44% reduction), affective pain (32% reduction), and sensory pain (27% reduction) were significantly lower (*p* < 0.05) with the adjunctive virtual-reality treatment than with the control. Immersive VR did not result in a significant increase in maximal range of joint motion compared to the control condition (*p* = 0.21). However, there was a significant increase in maximal range of motion (mean increase of 6.8 degrees, *p* = 0.03) in the second treatment condition.
Lee et al. (2022) [30]	Korea. (Asia Oriental)	Mean 57.55 ± 7.55	Interventional (clinical trial)	*n* = 33	Robot-assisted gait training (RAGT) in burn patients by analysing the cerebral blood flow (CBF) in the prefrontal cortex.	RAGT: 30 min sessions, 10 times for 2 weeks, from Monday to Friday. 15 min sessions of VR application, with a 2 min break, and 15 min session without VR.	Functional near-infrared spectroscopy (fNIRS). Visual analogue scale (VAS).	The mean VAS pain scores were significantly lower (*p* < 0.05) in the experimental condition than in the control condition. Oxyhaemoglobin in the prefrontal lobe significantly increased when RAGT was performed with VR. The results of the analyses conducted on HbO2 in the PFC indicated a significant VR-related PFC activation during RAGT, as compared with the results in the control condition.

Note: RCT: randomized controlled trial. VR: virtual reality. TOTG: task-oriented training group. EG: experimental group. CG: control group. XboxG: Xbox group. ROM: range of motion. AROM: active range of motion. JTHFT: Jebsen hand function test. DHI: Duruoz hand index. COPM: Canadian Occupational Performance Measure. GRS: graphic rating scale. VAT: visual analogue thermometer. VAS: visual analogue scale. PSC-C: Pain Catastrophizing Scale for Children. RAGT: robot-assisted gait training. fNIRS: functional near-infrared spectroscopy.

**Table 3:** Results of the methodological quality assessment with the Physiotherapy Evidence Database Scale (PEDro).

Criteria													
Studies	1	2	3	4	5	6	7	8	9	10	11	Total	Score Interpretation
Yohannan et al. [20]	Y	Y	N	Y	N	N	Y	Y	Y	Y	Y	7	Good
Lozano et al. [24]	Y	N	N	Y	N	N	N	Y	N	Y	Y	5	Average
Radwan et al. [31]	Y	Y	N	Y	N	N	N	Y	Y	Y	Y	6	Good
Kamel et al. [17]	Y	Y	Y	Y	N	N	Y	Y	Y	Y	Y	8	Good
Yoltani et al. [25]	Y	Y	N	Y	N	N	N	Y	Y	Y	Y	6	Good
Carrougher et al. [26]	Y	Y	N	Y	N	N	N	Y	Y	Y	Y	6	Good
Faber et al. [27]	Y	N	N	N	N	N	N	Y	Y	Y	Y	4	Average
Hoffman et al. [28]	Y	N	N	Y	N	N	N	Y	Y	Y	Y	5	Average
Schmitt et al. [29]	Y	N	N	Y	N	N	N	Y	Y	Y	Y	5	Average
Lee et al. [30]	Y	N	N	Y	N	N	N	Y	Y	Y	Y	5	Average

Note: Y: met criteria; N: did not meet criteria. Eligibility criteria specified; 2. Random assignment; 3. Concealed assignment; 4. Similar groups at baseline; 5. Blinding of all subjects; 6. Blinding of all therapists; 7. Blinding of all evaluators; 8. Follow-up of more than 85% of subjects; 9. Intention-to-treat analysis; 10. Between-group statistical analysis; 8. Follow-up of more than 85% of subjects; 9. Intention-to-treat analysis; 10. Between-group statistical comparisons; 11. Point measures and measures of variability are given for at least one key outcome.

**Table 4:** Risk of bias.

Criteria							
Studies	1	2	3	4	5	6	7
Yohannan et al. [20]	?	?	-	-	+	+	+
Lozano et al. [24]	-	-	-	-	+	+	+
Radwan et al. [31]	+	-	?	?	+	+	+
Kamel et al. [17]	+	+	-	+?	+	+	+
Soltani et al. [25]	?	?	-	?	-	+	+
Carrougher et al. [26]	?	?	-	-	+	+	+
Faber et al. [27]	-	?	-	-	+	+	+
Hoffman et al. [28]	?	?	-	?	+	+	+
Schmitt et al. [29]	?	?	-	-	+	+	+
Lee et al. [30]	-	-	-	-	+	+	+

+ = “Low risk” of bias; - = “High risk” of bias; ? = “Unclear risk” of bias; N/A = not applicable. 1 = random sequence generation (selection bias). 2 = allocation concealment (selection bias). 3 = blinding of participants and staff (implementation bias). 4 = blinding of outcome assessment (detection bias). 5 = incomplete outcome data (attrition bias). 6 = selective information (information bias). 7 = other biases.
